# Student Stress, Coping, and APPE Readiness at Two Public Institutions before and during the Pandemic

**DOI:** 10.3390/pharmacy12040121

**Published:** 2024-08-05

**Authors:** Tram B. Cat, Shareen Y. El-Ibiary, Kelly C. Lee

**Affiliations:** 1 UC San Francisco School of Pharmacy, San Francisco, CA 94143, USA; 2 College of Pharmacy—Glendale Campus, Midwestern University, Glendale, AZ 85308, USA; selibi@midwestern.edu; 3 UC San Diego Skaggs School of Pharmacy and Pharmaceutical Sciences, La Jolla, CA 92093, USA; kellylee@health.ucsd.edu

**Keywords:** student well-being, stress, COPE, professional development, pharmacy practice readiness, pandemic

## Abstract

The coronavirus disease 2019 (COVID-19) pandemic significantly impacted pharmacy students’ education and well-being. The primary aim of this study was to evaluate the effects of the pandemic on students’ perceived stress by comparing third- and fourth-year students from the pre-pandemic Class of 2019 with mid-pandemic Class of 2021 at two public institutions. Secondary aims were to evaluate the pandemic effects on students’ academic and professional development skills and practice readiness. The Perceived Stress Scale (PSS) and the Brief Coping Orientation to Problems Experienced (COPE) scale were used to measure student well-being. Students’ self-rated problem-solving, time management, and study skills were used to measure their academic and professional development; practice readiness was measured using students’ self-rated confidence levels. PSS scores were significantly higher in mid-pandemic than pre-pandemic students, and the Brief COPE avoidant coping subscale differed between pre-pandemic and mid-pandemic students. No differences were found in any academic and professional development skills between the pre- and mid-pandemic students, and there were significant improvements in student confidence levels for practice readiness among the mid-pandemic students. In conclusion, the pandemic appeared to affect students’ stress and avoidant coping mechanism but had variable effects on academic and professional development and practice readiness.

## 1. Introduction

Well-being and stress among pharmacy students have been at the forefront of pharmacy education. Particularly after the coronavirus disease 2019 (COVID-19) pandemic, there have been increasing concerns for pharmacy student well-being and stress. The promotion of student well-being and resilience has been a significant focus for the American Association of Colleges of Pharmacy (AACP), gaining such importance that it has also been explicitly highlighted in American Council on Pharmaceutical Education (ACPE) standards. The 2025 ACPE draft standards refer frequently to the promotion of student well-being along with student success as a criterion of comprehensive student services provided to students [1]. The 2025 ACPE draft standards have also continued to require that all institutions offering the Doctor of Pharmacy (Pharm.D.) degree assess students’ Advanced Pharmacy Practice Experience (APPE) readiness as part of the school’s assessment plan to measure student achievement at defined levels of their professional competencies [1]. While student well-being and practice readiness are clearly referenced in the ACPE standards, it is unknown how stress and well-being in pharmacy students affect their confidence and practice readiness. Various studies have investigated predictors of APPE readiness, such as performance in capstone courses [2,3], top drug competency exams [2], patient case classroom activity performance [4], student failure or unprofessionalism issues [5], and understanding of the patient workup process [6]. No study to our knowledge, however, has evaluated changes in student-perceived practice readiness before and during the pandemic. As many healthcare professionals and students had to adapt to changes in work and learning settings during the pandemic, pharmacy students were no exception. Changes and uncertainties during the pandemic likely induced stress, forcing students to use coping methods to adapt and manage stress [7]. Elharake et al. conducted a systematic review of mental health effects during the COVID-19 pandemic and found that stress among children and college students increased from 24% to 71% due to the pandemic [8]. Another study suggested that low advanced pharmacy practice experiences (APPEs) confidence may have been due to remote didactic and Introductory Pharmacy Practice Experiences (IPPEs) and changes in pre-APPE preparatory courses imposed by the pandemic [9]. On the other hand, studies by Pham et al. and Khosraviani et al. found no differences in perceived stress between pre-pandemic and mid-pandemic pharmacy students [10,11]. This study aims to evaluate pharmacy students’ self-perceived student stress, coping strategies, academic and professional development skills, and self-confidence in APPE practice readiness during pre- and mid-pandemic periods.

## 2. Materials and Methods

### 2.1. Design

This was a retrospective study of data collected at two public institutions as part of an annual student quality of life measurement initiative. The purpose was to collect and track students' well-being while they advanced through their respective programs. Electronic surveys were administered via Qualtrics at the beginning of the first year (P1) and then annually at the end of second year (P2), third year (P3) and fourth year (P4). For the purposes of this study, student responses from the end of years P3 and P4 for the pre-pandemic Class of 2019 and mid-pandemic Class of 2021 were used.

### 2.2. Instruments

The surveys included two validated scales (Perceived Stress Scale (PSS) and Brief Coping Orientation to Problems Experienced (COPE)) and two scales (academic and professional development, APPE practice readiness) developed through joint collaborations between the two institutions. The PSS was used because it has been proven to be valid and reliable for use in university student populations [12,13] and has also been correlated with other well-being domains [14,15,16]. The perceived stress of students was measured using a validated 10-item PSS [17] that captured perceived stress among respondents during the last month using a 5-point Likert scale (0 = never, 1 = almost never, 2 = sometimes, 3 = fairly often, 4 = very often for questions 1, 2, 3, 6, 9, and 10, and reverse scoring for questions 4, 5, 7, and 8). The mean PSS scores were calculated using the sum of all items and dividing by the number of items [17]. To assess how students respond to stressful events, the Brief COPE scale was used, which is an abbreviated 28-item version of the COPE inventory with a 4-point Likert scale (1 = I haven’t been doing this at all, 2 = I’ve been doing this a little bit, 3 = I’ve been doing this a medium amount, 4 = I’ve been doing this a lot) [18]. The scale produces a respondent’s primary coping style on three subscales: problem-focused coping, emotion-focused coping, and avoidant coping. To measure students’ academic and professional development skills, students rated their perceived general problem-solving skills, time management skills, and study skills using a 5-point Likert scale (1 = very poor, 2 = poor, 3 = fair, 4 = good, 5 = very good) (three questions total). We also measured students’ ratings of their self-confidence in APPE practice readiness in five areas: being an effective member on a patient care team, being an excellent practicing pharmacist, their leadership skills, their scientific investigation skills, and their life-long learning skills, using a 5-point Likert scale (1 = not at all confident, 2 = a little confident, 3 = moderately confident, 4 = mostly confident, 5 = very confident) (five questions total). 

### 2.3. Data Collection

**Figure 1:**
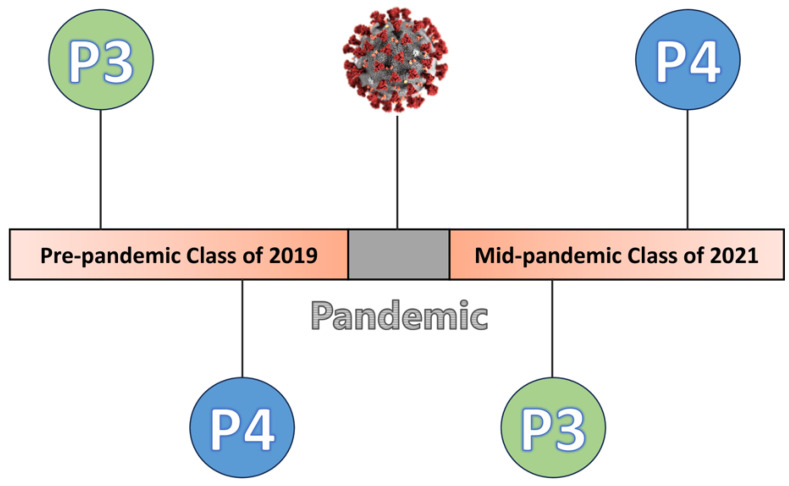
Pharmacy Student P3 and P4 Year Pandemic Timeline.

Student survey responses from pre-pandemic Class of 2019 and mid-pandemic Class of 2021 were evaluated at end of year for P3 and P4 students at both institutions as part of a quality assessment of student well-being. End of year responses for P3 and P4 students were collected during academic years 2018–2019 (pre-pandemic) and 2020–2021 (mid-pandemic) (Figure 1). The surveys were administered via Qualtrics (Provo, UT, USA). The electronic survey included demographic questions about students’ class, students’ relationship status (married/domestic partner), and number of dependents. Students who did not respond to all questions for the PSS were excluded from the analysis. The study received approval and exempt status from UCSD and UCSF Offices of IRB Administration, respectively.

The primary outcomes of the study were to compare PSS scores and Brief COPE subscales (problem-focused, emotion-focused, and avoidant coping) between two study groups, pre-pandemic Class of 2019 and mid-pandemic Class of 2021. Secondary outcomes included a comparison of academic and professional development skills as well as self-confidence in APPE practice readiness between pre-pandemic Class of 2019 and mid-pandemic Class of 2021.

### 2.4. Data Analysis and Interpretation

Descriptive statistics were used (number, percent, mean, standard deviation (SD)) to characterize the study groups. The primary outcome (mean PSS score between the two study groups) was analyzed using the independent *t*-test, where a *p*-value < 0.05 was considered statistically significant. Secondary outcomes for self-rated academic and professional development skills and APPE practice readiness confidence were compared between the two study groups using the Mann–Whitney U test. Correlation between PSS and academic and professional development skills and APPE practice readiness were analyzed using Pearson correlation tests, where a *p*-value < 0.05 considered statistically significant. All analyses were conducted using IBM SPSS Statistics Version 28.0 (Armonk, NY, USA).

## 3. Results

During the years sampled, response rates for pre-pandemic Class of 2019 and mid-pandemic Class of 2021 were 67.3% (*n* = 249) and 67.6% (*n* = 250), respectively. Of the total of 499 students from both pre- and mid-pandemic classes, there were 275 students who were in their P3 year and 224 students who were in their P4 year. Of these, 66 (13.2%) students were married, and 430 (86.2%) students were not married (three students did not respond). The majority of students did not have dependents (*n* = 471, 94.4%). 

### 3.1. Perceived Stress

Students at the end of their P3 and P4 years in the mid-pandemic Class of 2021 had a significantly higher PSS total score compared to the pre-pandemic Class of 2019 (Table 1). There was also a significant difference in PSS scores in P3 students between pre-pandemic Class of 2019 and mid-pandemic Class of 2021; P3 students in the mid-pandemic Class of 2021 had a higher PSS score than P3 students in the pre-pandemic Class of 2019 (19.0 ± 6.30 and 17.5 ± 6.32, *p* = 0.048, respectively). There was no difference in PSS scores among P4 students between pre-pandemic Class of 2019 and mid-pandemic Class of 2021 (*p* = 0.241). There were no differences between P3 and P4 students within the pre-pandemic Class of 2019 and mid-pandemic Class of 2021.

### 3.2. Brief COPE

Brief COPE summative scores for problem-focused, emotion-focused, and avoidant coping subscales were compared by class and by year level of pharmacy students. While there were no differences in the problem-focused and emotional-focused Brief COPE subscales between pre-pandemic Class of 2019 and mid-pandemic Class of 2021, there was a significant difference in the avoidant coping subscale (Table 2). When explored further, the mean scores for avoidant coping were also higher for P3 students in mid-pandemic Class of 2021 compared to P3 students in pre-pandemic Class of 2019 (14.1 ± 3.62 vs. 12.9 ± 2.69, *p* = 0.001), respectively. No differences in any of the Brief COPE subscales were found among P4 students between pre-pandemic Class of 2019 and mid-pandemic Class of 2021, nor between P3 and P4 students within pre-pandemic Class of 2019 and mid-pandemic Class of 2021.

### 3.3. Academic and Professional Development Skills

There were no differences in any of the academic and professional development skills between the pre-pandemic Class of 2019 and mid-pandemic Class of 2021 at the end of years P3 and P4 (Table 3). Additional evaluation of these skills at the P3 and P4 year levels between pre-pandemic Class of 2019 and mid-pandemic Class of 2021 as well as the comparison between P3 and P4 students within the Classes of 2019 and 2021 also showed no differences.

### 3.4. Confidence Level for APPE Practice Readiness 

When evaluating students’ confidence levels for APPE practice readiness, mid-pandemic Class of 2021 students’ confidence levels increased significantly from year P3 to P4 across all five areas: being an effective member of a patient care team, being an excellent practicing pharmacist, leadership, scientific investigation, and life-long learning (Table 4). Pre-pandemic Class of 2019 students’ confidence levels, however, only increased significantly from year P3 to P4 in being an effective member of a patient care team (Table 4). Upon further evaluation, a significant decline in confidence levels was observed among P3 students in the pre-pandemic Class of 2019 vs. mid-pandemic Class of 2021 in the following areas: being an effective member of a patient care team (150.03 vs. 121.06, *p* = 0.001), being an excellent practicing pharmacist (146.65 vs. 124.91, *p* = 0.018), and leadership skills (144.87 vs. 126.95, *p* = 0.049). There was no difference in P4 students’ confidence for APPE practice readiness between pre-pandemic Class of 2019 and mid-pandemic Class of 2021.

### 3.5. Correlation between PSS and Academic and Professional Development Skills

To determine whether higher perceived stress affected academic and professional development skills, correlation analyses were conducted. Significant negative correlations were observed between PSS scores and students’ self-ratings of academic and professional development skills among the pre-pandemic Class of 2019 and mid-pandemic Class of 2021 (Table 5). Additional analyses at the P3 and P4 year level showed there was an overall stronger negative correlation between PSS scores and problem-solving, time management, and study skills for P3 pre-pandemic Class of 2019 compared to P3 mid-pandemic Class of 2021. For P3 students in the mid-pandemic Class of 2021, negative correlations between PSS scores and the academic and professional development areas were only significant for study skills. In contrast, for P4 students in the pre-pandemic Class of 2019, time management and study skills were strongly negatively correlated with PSS scores (r = −0.412, *p* < 0.001 and r = −0.301, *p* = 0.002, respectively) compared to the P4 mid-pandemic Class of 2021, whose PSS scores were only correlated with problem-solving skills (r = −0.250, *p* = 0.006). 

### 3.6. Correlation between PSS and APPE Practice Readiness

Significant negative correlations were found between students’ PSS scores and confidence levels for practice readiness for both pre-pandemic Class of 2019 and mid-pandemic Class of 2021 in all areas, except in leadership skills for mid-pandemic Class of 2021 (r = −0.124, *p* = 0.052) (Table 5). For P3 students in the mid-pandemic Class of 2021, correlations between PSS scores and practice readiness were only significant for lifelong learning skills (r = −0.227, *p* = 0.010). Confidence levels for practice readiness among P4 students were negatively correlated with PSS scores in all areas for both pre-pandemic and mid-pandemic classes except for leadership skills (r = −0.183, *p* = 0.065) in the pre-pandemic Class of 2019 students.

## 4. Discussion

Based on the results of our study, the pandemic appeared to affect students’ stress and avoidant coping mechanisms. However, the effects of the pandemic were variable in students’ academic and professional development and practice readiness. It is important to note that given the self-reported nature of our survey, we approached the interpretation of our study results with caution, understanding that response bias might have influenced our findings.

### 4.1. Stress and Coping

When comparing the perceived stress between the two student cohorts, the higher perceived stress level was seen in the mid-pandemic Class of 2021. The higher level of mid-pandemic Class of 2021 compared to pre-pandemic Class of 2019 students may be attributed to the significant societal impact of the COVID-19 pandemic. The P3 students in the mid-pandemic Class of 2021, who were surveyed in Spring of 2020 during the height of the pandemic, had significantly higher PSS levels compared to the P3 students in the pre-pandemic Class of 2019. The findings in this current study are supported by Cat et al., where 62% of survey respondents from Class of 2021 reported COVID-19 as a stress factor [19]. Additionally, as seen in previous studies of medical and dental students and undergraduate college students [7,8,20,21,22], it was not a surprise to also observe increased stress levels among pharmacy students in the mid-pandemic Class of 2021 compared to the pre-pandemic Class of 2019. The current study findings contrast with the study by Pham et al.; however, it is important to note that Pham et al. evaluated P1 students at the end of year rather than P3 and P4 students at the end of year [10]. The types of stress factors experienced by students in their P3 and P4 years may be different from those of P1 students and could be influenced by concerns for APPEs [19] and postgraduate plans [23].

Interestingly, both pre-pandemic Class of 2019 and mid-pandemic Class of 2021 students were found to have stress levels that were higher than the normative mean PSS scores (14.2 ± 6.2) for those aged 18–29 [24]. It is unclear why pharmacy students would have increased perceived stress levels, but this possibly could be explained by students’ concern for post-graduate plans, as they often need to prepare and interview for jobs or residencies. Given the increasing competitiveness of residency matches and the oversupply of PharmD graduates for pharmacist job openings, it is no surprise that these issues are stress factors for student pharmacists [25,26]. Consistent with this finding was a study by Votta and Beanu that identified the job market as a more frequently cited stress factor among students in the later years of pharmacy school [23]. Programs may want to consider working with students to help alleviate stress that could be related to employment concerns.

Along with the increased PSS total scores, mid-pandemic Class of 2021 students were also found to have higher avoidant coping behaviors compared to the pre-pandemic Class of 2019 students. A possible explanation for this observation could be due to higher physical and cognitive disengagement because of the COVID-19 pandemic. Findings from Cat et al. showed moderate depersonalization (i.e., an unfeeling and impersonal response toward recipients of one’s care or service) [27] scores in mid-pandemic Class of 2021 students in Spring of 2020, which underscores the potential impact that the pandemic may have had on students [19].

### 4.2. Academic and Professional Developmental Skills

It is interesting to note that the COVID-19 pandemic did not appear to affect the mid-pandemic students’ perceived academic and professional developmental skills either as a class or professional year in school. This is consistent with a study by Zavaleta et al. that showed no relationship between stress and academic performance among engineering students during the pandemic [28]. A study by Hettinger et al., while showing that pre-pandemic pharmacy students reported higher perceived stress levels before and after performance-based assessments compared to students during the pandemic, found no correlation with actual performance [29]. In a qualitative study, pharmacy students expressed positive impacts on their learning due to the pandemic [30]. They expressed factors such as having “more time for themselves, family friends” and “less travel commuting” as overall positively contributing to their studies. In a survey study of students at Historically Black Colleges and Universities (HBCUs), the investigators reported that students did not find COVID-19 restrictions impacting their ability to learn and retain information [31]. It appears that despite the pandemic, students displayed resilience toward their academic and professional development skills. Based upon the conflicting results between the existing literature [32,33] and the current study, it is unclear exactly what role the PSS has in measuring students’ ability to problem-solve, manage their time, and study.

### 4.3. Confidence in APPE Practice Readiness

Interestingly, mid-pandemic Class of 2021 students had more observed increases in self-reported confidence in practice readiness when transitioning from year P3 to P4 than students in the pre-pandemic Class of 2019. This may be due to a few reasons. The pandemic may have provided more opportunities for P4 year mid-pandemic Class of 2021 students to play a greater role as part of healthcare teams along with their preceptors. In late 2020 and early 2021, pharmacists played a large and integral part in the public health initiative to vaccinate individuals against COVID-19 [34]. At the start of the pandemic in 2020, there were many societal restrictions, curfews, business shutdowns, and remote learning, which may have affected student pharmacists’ outlooks on their education and profession [35,36,37]. In 2021, when P4 students in the mid-pandemic Class of 2021 were surveyed, pharmacists were seen as key healthcare providers at the forefront of vaccination campaigns [34,38,39]. P4 students in the mid-pandemic Class of 2021 were involved in various vaccine clinics that gave patients a positive perception of pharmacists [40,41]. These interactions and the modeling displayed by pharmacist preceptors with vaccination efforts may have helped to increase the confidence of students from years P3 to P4 in the mid-pandemic Class of 2021. Further, these experiences may help to explain the differences seen in P3 students between mid-pandemic Class of 2021 and pre-pandemic Class of 2019, as there were so many uncertainties about the pandemic in Spring 2020 for P3 students in mid-pandemic Class of 2021. While it is expected that perceived practice readiness would increase from the P3 to P4 year in pharmacy school, the observed difference was much greater in mid-pandemic Class of 2021, which suggests that the pandemic may have contributed to this observed difference.

Another interesting finding from our study was the decline in self-reported confidence levels observed in three of the five areas of practice readiness when pre- vs. mid-pandemic classes for P3 students were compared. A possible explanation for this could be that those three areas of practice readiness (being an effective member of a patient care team, being an excellent practicing pharmacist, and belief in their leadership skills) required some level of in-person and hands-on experience for students to gain confidence and feel APPE-ready [9,42,43]. Since the mid-pandemic Class of 2021 students may have had Introductory Pharmacy Practice Experiences (IPPEs) switched to remote experiential learning, this could have hindered their ability to develop non-technical skills, sometimes referred to as soft skills, that are inherent when observing and being physically present as preceptors model their work and interact with others in their practice environment [9,42,43]. The lack of difference in self-reported confidence levels among P4 students between pre- vs. mid-pandemic classes may have been due to students’ ability to obtain hands-on experiences during their APPE year, where their knowledge and skills (technical and non-technical) were reinforced through continuous application in real-life practice settings, regardless of the pandemic [43,44].

### 4.4. Correlations between PSS and Academic and Professional Development Skills and APPE Practice Readiness 

The negative correlations observed in pre-pandemic Class 2019 and mid-pandemic Class of 2021 between PSS and academic and professional development skills suggest that high perceived stress negatively impacted students’ problem-solving, time management, and study skills. This finding is supported by a study on university students that showed a significant negative relationship exists between students’ self-efficacy and academic burnout, with stress being an important component of burnout [32]. Similarly, negative correlations were found between PSS and confidence levels for all areas of APPE practice readiness in both pre-pandemic Class of 2019 and mid-pandemic Class of 2021 students, except for leadership skills in mid-pandemic Class of 2021. Because mid-pandemic Class of 2021 students may have felt there was a “call to duty” to be part of a profession that was leading efforts in vaccinating patients [41], their leadership skills may have been impervious to stress.

Another interesting finding was the overall stronger negative correlations observed between PSS scores and academic and professional development in P3 pre-pandemic Class of 2019 compared to P3 mid-pandemic Class of 2021 students. With the mid-pandemic Class of 2021 P3 students having remote learning in didactic and sometimes in IPPEs, more time spent with family, friends and for themselves as well as less time for commuting [30] may have shielded them from the typical stress that normally could be a factor for students, and that was likely experienced by P3 pre-pandemic Class of 2019. When looking at academic and professional development and APPE practice readiness, similarly strong negative correlations were observed between P4 mid-pandemic Class of 2021 and P4 pre-pandemic Class of 2019 students. This finding may be due to the common stressors, such as post-graduate planning [19,23], that are often experienced by P4 students and could affect their academic and professional development skills and practice readiness, regardless of the pandemic.

### 4.5. Limitations

There were several limitations to this study. As commonly found in survey studies, we had missing responses that may have affected our study results. Additionally, since our survey items required respondents to self-report their perceptions, the items could have been interpreted differently, thereby, introducing response bias into our study results. Further, due to the scope and design of the study, we were unable to determine whether a trend existed with student stress over time or if it was specific to the pandemic. Also, the sample was limited to students from two schools of pharmacy belonging to the same University system located in the state of California. Pharmacists’ experiences and duties in California, as well as pandemic-related restrictions, may have differed from those in other states; therefore, these external factors may have affected students’ reported perceptions and experiences. To maintain the confidentiality of our respondents, we intentionally collected limited demographics information. Based on all the aforementioned limitations, our study’s findings may not be generalizable to other schools or colleges of pharmacy.

## 5. Conclusions

Pharmacy students who graduated a year after the start of the pandemic had higher perceived stress and avoidant coping behavior compared to students who graduated pre-pandemic. There were strong negative correlations between perceived stress and academic and professional development and practice readiness in pre-pandemic and mid-pandemic classes. While the pandemic potentially boosted student pharmacists’ confidence in seeing themselves as integral members of healthcare teams, its full impact, including that on stress management abilities, however, remains unclear for future generations. We feel our study's findings have shed more light on students’ perceived stress and coping behaviors and hope they will help other schools of pharmacy evaluate how they can better enable their students to be resilient and thrive, even when a crisis is encountered. Further studies, however, are needed to explore whether the current findings can be generalizable to students from other schools or colleges of pharmacy.

## Figures and Tables

**Table 1:** Overall perceived stress scale (PSS ^a^) of pre-pandemic Class of 2019 ^b^ vs. mid-pandemic Class of 2021 ^b^.

	Class of 2019 *n* = 249 Mean (SD)	Class of 2021 *n* = 250 Mean (SD)	*p*-Value
PSS	17.3 (6.37)	18.5 (6.73)	0.036

^a^ PSS: 5-point Likert scale (0 = never, 1 = almost never, 2 = sometimes, 3 = fairly often, 4 = very often for questions 1, 2, 3, 6, 9, and 10, and reverse scoring for questions 4, 5, 7, and 8). ^b^ Students at end of their 3rd and 4th year of pharmacy school.

**Table 2:** Overall brief COPE ^a,b^ of pre-pandemic Class of 2019 ^c^ vs. mid-pandemic Class of 2021 ^c^.

	Class of 2019 *n* = 248 Mean (SD)	Class of 2021 *n* = 246 Mean (SD)	*p*-Value
Brief COPE Subscales			
Problem-Focused	22.0 (5.16)	21.7 (4.89)	0.484
Emotion-Focused	27.4 (5.82)	28.0 (6.11)	0.270
Avoidant Coping	13.0 (2.97)	13.7 (3.54)	0.017

^a^ COPE: Coping Orientation to Problems Experienced. ^b^ Brief COPE scale: 4-point Likert scale (1 = I haven’t been doing this at all, 2 = I’ve been doing this a little bit, 3 = I’ve been doing this a medium amount, 4 = I’ve been doing this a lot). ^c^ Students at end of their 3rd and 4th year of pharmacy school.

**Table 3:** Academic and professional development skills ^a,b^ of pre-pandemic Class of 2019 ^c^ vs. mid-pandemic Class of 2021 ^c^.

	Class of 2019 *n* = 248	Class of 2021 *n* = 246	*p*-Value
Academic and Professional Development Skills			
Problem-Solving Skills	243.31	251.72	0.466
Time Management Skills	247.43	247.57	0.991
Study Skills	251.79	243.18	0.476

^a^ Academic and professional development skills: 5-point Likert scale (1 = very poor, 2 = poor, 3 = fair, 4 = good, 5 = very good). ^b^ Displays mean rankings of the Likert scale items based on Mann–Whitney analyses. ^c^ Students at end of their 3rd and 4th year of pharmacy school.

**Table 4:** Confidence level ^a,b^ for APPE ^c^ practice readiness of pre-pandemic Class of 2019 and mid-pandemic Class of 2021 between P3 ^d^ and P4 ^e^ students.

	Class of 2019			Class of 2021		
	P3 *n* = 145	P4 *n* = 103	*p*-Value	P3 *n* = 127	P4 *n* = 119	*p*-Value
Confidence Level for APPE Practice Readiness						
Being an effective member of a patient care team	117.30	134.64	0.048	104.02	144.29	<0.001
Being an excellent practicing pharmacist	118.98	132.27	0.132	111.67	136.13	0.005
Leadership skills	121.29	129.02	0.378	115.12	132.44	0.044
Scientific investigation skills	121.11	129.27	0.361	111.91	135.87	0.006
Life-long learning skills	123.10	126.48	0.698	113.71	133.95	0.019

^a^ Confidence level scale: 5-point Likert scale (1 = not at all confident, 2 = a little confident, 3 = moderately confident, 4 = mostly confident, 5 = very confident). ^b^ Displays mean rankings of the Likert scale items based on Mann–Whitney analyses. ^c^ APPE: Advanced Pharmacy Practice Experiences. ^d^ P3: students in 3rd year of pharmacy school. ^e^ P4: students in 4th year of pharmacy school.

**Table 5:** Correlation between perceived stress scale (PSS ^a^) and academic and professional development skills and confidence level for APPE practice readiness in pre-pandemic Class of 2019 ^b^ and mid-pandemic Class of 2021 ^b^.

	Class of 2019 *n* = 249 ^c^		Class of 2021 *n* = 250 ^d^	
	r-Value ^f^	*p*-Value	r-Value ^f^	*p*-Value
Academic and Professional Development Skills ^e^				
Problem-Solving Skills	−0.204	0.001	−0.205	0.001
Time Management Skills	−0.368	<0.001	−0.178	0.005
Study Skills	−0.241	<0.001	−0.228	<0.001
Confidence Level ^g^ for APPE Practice Readiness				
Being an effective member of a patient care team	−0.255	<0.001	−0.232	<0.001
Being an excellent practicing pharmacist	−0.234	<0.001	−0.209	<0.001
Leadership skills	−0.195	0.002	−0.124	0.052
Scientific investigation skills	−0.229	<0.001	−0.150	0.019
Life-long learning skills	−0.256	<0.001	−0.230	<0.001

^a^ PSS: 5-point Likert scale (0 = never, 1 = almost never, 2 = sometimes, 3 = fairly often, 4 = very often for questions 1, 2, 3, 6, 9, and 10, and reverse scoring for questions 4, 5, 7, and 8). ^b^ Students at end of their 3rd and 4th year of pharmacy school. ^c^ *n* = 249 for PSS; *n* = 248 for academic and professional development skills and APPE practice readiness items. ^d^ *n* = 250 for PSS; *n* = 246 for academic and professional development skills and APPE practice readiness items for all others. ^e^ Academic and Professional Development Skills scale: 5-point Likert scale (1 = very poor, 2 = poor, 3 = fair, 4 = good, 5 = very good). ^f^ Nonparametric correlations displayed. ^g^ Confidence Level scale: 5-point Likert scale (1 = not at all confident, 2 = a little confident, 3 = moderately confident, 4 = mostly confident, 5 = very confident).

## Data Availability

The datasets presented in this article are not readily available because the data are part of an ongoing quality improvement process at the institutions. Requests to access the datasets should be directed to the corresponding author.
